# Early-Onset Respiratory Muscle Paralysis in Crotalic Envenomation: A Case Study

**DOI:** 10.1590/0037-8682-0374-2023

**Published:** 2023-11-10

**Authors:** Juliana Sartorelo Almeida, Felipe Carvalhaes Possas, Adebal de Andrade, Samir de Oliveira Sauzen, Rodrigo Ganem Sugino

**Affiliations:** 1 Hospital João XXIII, Centro de Informação e Assistência Toxicológica de Minas Gerais, Belo Horizonte, Minas Gerais, Brasil.; 2 Universidade Federal de São Paulo, São Paulo, Brasil.

**Keywords:** Crotalus, Respiratory failure, Antivenom

## Abstract

Crotalic envenomation is responsible for approximately 8%-13% of ophidism cases in Brazil, yet it is associated with the highest mortality among snakes. We describe the case of a patient bitten by a rattlesnake who developed ventilatory muscle paralysis within hours after envenomation. While diaphragmatic paralysis is a rare late neurotoxic event following crotalic envenomation, in this case, paralysis occurred early but was rapidly reversed after antivenom administration. This report discusses potential contributing factors based on a comprehensive literature review.

## INTRODUCTION

*Crotalus* spp., commonly known as rattlesnakes, are among the primary causative agents of ophidic envenomation, especially in North America^1^. *Crotalus durissus*, the predominant species in Latin America, belongs to the *Viperidae* family and is distributed across Brazil, primarily in dry and semiarid regions^1^ ^,^ ^2^.

Crotalic envenomations account for 8%-13% of all ophidism cases in Brazil, with 10%-14% being considered severe cases associated with high morbidity and mortality^2^. In 2020, Brazil reported 2,813 crotalic bites and 22 deaths through the National System for Notification of Diseases (SINAN), with 657 (23%) occurring in the state of Minas Gerais, resulting in a mortality rate of 0.78%^3^.

The snakes' venom contains four toxins: crotoxin, crotamine, convulsin, and Gyrotoxin^4^. Crotoxin, accounting for 50% of the molecular weight, consists of two non-identical subunits, Crotoxin A and Crotoxin B^5^ ^-^ ^7^. The synergistic action of these fractions induces neurotoxicity by inhibiting presynaptic acetylcholine release, potentially affecting skeletal muscles. Crotamine, a polypeptide composed of 42 amino acids, causes muscle fiber depolarization, leading to muscle contraction and paralysis. Convulxins, C-type lectins, induce cardiovascular and pulmonary disorders, while Giroxin, a serine protease, contributes to haemotoxicity affecting fibrinogen and fibrin formation^5^ ^-^ ^7^. Crotoxins and crotamine are primarily responsible for the myotoxic effects of the venom, leading to severe rhabdomyolysis, which can result in kidney damage and subsequent failure, a major cause of envenomation-related deaths^7^.

Specific antivenom serum is available to treat clinical manifestations associated with the neurotoxic, nephrotoxic, myotoxic, and coagulant effects of the venom, and it should be administered as promptly as possible^8^. Currently, in Brazil, antivenom is exclusively produced and distributed for healthcare use by the Butantan Institute, the largest producer of vaccines and antivenoms in Latin America. Antivenom is distributed to healthcare institutions nationwide, following the guidelines of the Ministry of Health.

This report describes the case of a patient bitten by a rattlesnake who rapidly developed signs of respiratory muscle paralysis, necessitating ventilatory support. This is the first case report of early respiratory failure and rapid recovery following the administration of antivenom for rattlesnake envenomation in Brazil. Our search and literature review utilized data sources from the Cochrane Library, LILACS, SciELO, MEDLINE, PubMed, and PMC (PubMed Central). This study received approval from the institutional ethics committee.

## CASE DESCRIPTION

**FIGURE 1:**
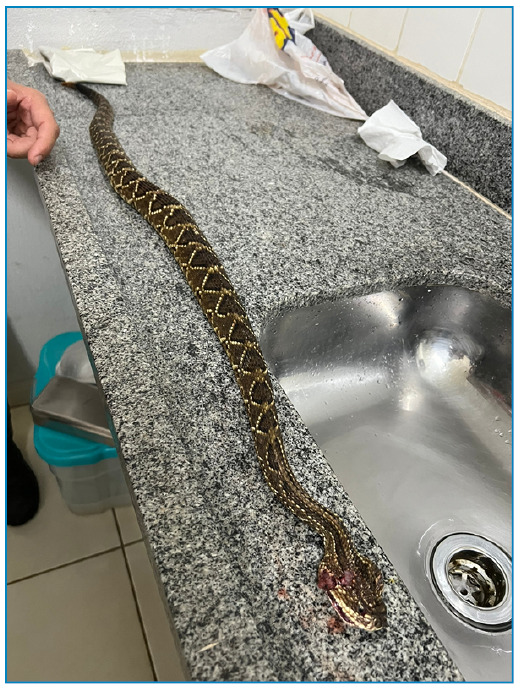
The *Crotalus durissus terrificus* specimen was offered by the patient. **Credits:** Felipe Carvalhaes.

**FIGURE 2:**
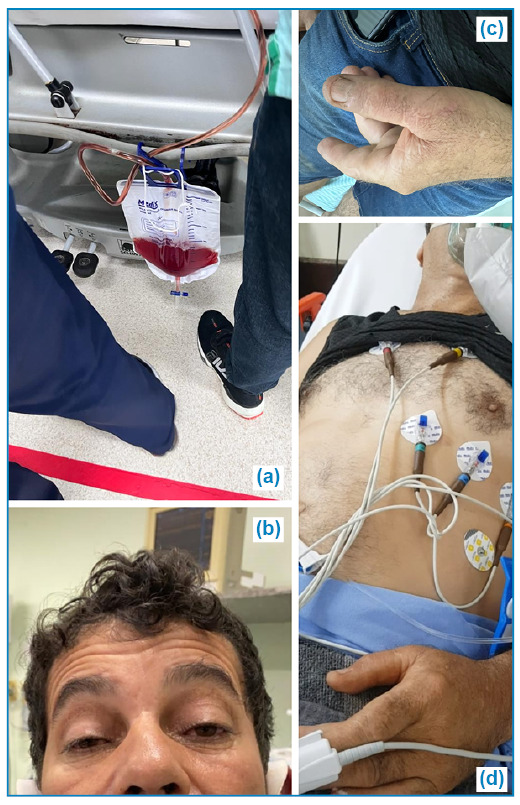
**(a)** Appearance of the urine on admission; **(b)** Ptosis present at the time of discharge **(c)** Location of the bite; **(d)** Patient's condition on admission.

A 53-year-old male patient without comorbidities, was bitten by a *Crotalus durissus terrificus* serpent (Figure 1) on his left thumb at home (Figure 2). He sought emergency care in his town 30 minutes after the bite, presenting with local pain, myalgia, blurred vision, malaise, and dyspnea. He received 1000 ml of 0.9% saline solution for hydration, analgesia, and a delayed urinary catheter for diuresis quantification. A Reference Center for animal envenomation was consulted, and the case was classified as severe. Immediate administration of 20 vials of crotalid antivenom was recommended, but it was unavailable, necessitating the patient's transfer to the Reference Center. Initial laboratory examinations in the originating unit revealed an uncoagulable blood and no abnormalities in renal function, CPK, or hemogram (Table 1).

**TABLE 1:** Patients laboratory evolution during hospitalization.

Laboratory Tests								
Date	Urea	Creatinine	Fibrinogen	INR	APTT	GOT	GPT	CPK
Jan 25	36	1.10	<35	>10	>120	1784	207	14966
Jan 26	42	1.25	65	1.49	25.2			65566
Jan 27	32	0.78	166	1.1				60803
Jan 28	37	0.99						11926
Jan 30	37	0.88				42	36	1324

*INR: international normalized ratio; **APTT:** Activated Partial Thromboplastin Clotting Time.

Upon arrival at the reference hospital four hours after the snakebite, the transportation team reported that the patient complained of worsening dyspnea, which progressed concurrently with the deterioration of the respiratory pattern during transportation between healthcare units. The patient was admitted to the emergency room with a non-rebreather mask at 5 L/min, complaining of dyspnea and pain at the sting site (Figure 2). In the initial evaluation, the patient presented with sweating and tachydyspnea, along with significant bilateral palpebral ptosis. There were no pulmonary or cardiac auscultation abnormalities, and the urine color was slightly reddish (Figure 2). Blood pressure was 182/120 mmHg, heart rate was 117 beats/min, respiratory rate was 36 beats/min, oxygen saturation was 99%, and capillary glycemia was 128 mg/dL.

Immediate administration of 20 vials of crotalid antivenom produced by the Butantan Institute was performed without complications. As the patient exhibited paradoxical breathing, characterized by the absence of diaphragmatic movement, use of abdominal musculature, and minimal accessory musculature movement, orotracheal intubation was performed due to the high risk of muscle fatigue four hours after envenomation. Gasometry indicated respiratory acidosis.

Intubation was accomplished with fentanyl, etomidate, and rocuronium via direct laryngoscopy. The patient received volume-controlled mechanical ventilation using minimal parameters.

The patient developed significant rhabdomyolysis (creatinine kinase peaked at 65,566 UI/l on the second day after envenomation), incoagulable blood, and a creatinine peak of 1.25 mg/dL. Progressive laboratory improvement was observed following antivenom administration. The patient remained under sedation until muscular strength improved and the respiratory pattern normalized. Extubation was performed without complications approximately 16 hours after endotracheal intubation.

The patient was discharged from the hospital in good clinical condition on the sixth day after the bite (Figure 2). Palpebral ptosis persisted for 15 days after envenomationn. Following discharge, he received outpatient follow-up in his hometown until complete recovery from neurotoxicity. 

## DISCUSSION

Crotalic envenomation in Brazil is associated with a high mortality rate, primarily due to acute renal failure resulting from direct nephrotoxicity and myoglobinuria, a consequence of severe rhabdomyolysis^7^ ^,^ ^8^. Crotoxin, a potent presynaptic beta-neurotoxin, and phospholipase A2 inhibit acetylcholine release at the neuromuscular junction^5^ ^,^ ^10^. Neurotoxicity in crotalic envenomation typically manifests early, causing paralysis of the palpebral and oculomotor musculature^10^. The severe and early progression observed in this case is seldom reported in the literature. Several factors may contribute to this pathophysiology, such as a substantial venom dose, a high concentration of Crotoxin in the venom, or immediate intravascular inoculation^9^ ^-^ ^12^.

A study by Vital Brazil in 1966 administered crotoxin and non-purified venom to animals intravenously (IV), intramuscularly (IM), and subcutaneously (SC) to establish a toxic dose^4^. SC injection required a dose four times higher than IV injection to induce muscle paralysis, while IV injection had effects similar to curare-like substances. Major paralysis was observed only when large volumes of venom were injected, with diaphragmatic paralysis occurring relatively late. In dogs and cats, extensive muscle paralysis manifested 48-72 hours after administration^5^.

According to Amaral et al.^9^, crotoxin concentrations in the plasma of envenomated patients rapidly decrease after envenomation, suggesting rapid distribution to deep tissue compartments, such as muscles and neuroreceptors. Early progression to respiratory failure is rarely documented in the literature. De Root et al.^11^ explored this subject and concluded that the amount of venom a snake can inject correlates with its body size, estimated from the distance between the fang punctures. Furthermore, as the distance between puncture marks provides an estimate of the snake's size, it allows for an estimation of its size and the potential amount of venom injected^11^.

The favorable outcome in this case, with extubation occurring slightly over 12 hours after intubation, may be attributed to the efficacy of the Butantan Institute-produced antivenom serum. Several studies have demonstrated the effectiveness of specific antivenoms against *Crotalus durissus* ^7^ ^,^ ^9^ ^,^ ^12^. In a study by Amaral et al.^12^, plasma levels of total venom, crotoxin, and antivenom measured over time revealed the efficacy of antivenom treatment. Circulating venom and crotoxin were no longer detectable one hour after antivenom therapy, and high antivenom levels persisted for at least 24 hours post-treatment.

In this case, despite the severity of envenomation, the patient received specific serum therapy for an adequate duration, contributing to a favorable outcome with the resolution of respiratory failure in fewer than 24 hours.

## CONCLUSION

Although rarely documented in the literature, respiratory muscle paralysis can occur and is a serious manifestation requiring prompt identification and treatment^9^ ^,^ ^10^. Adequate treatment with a specific antivenom typically reverses the symptoms without sequelae^10^. Severely poisoned patients without timely access to intubation and mechanical ventilation are at risk of asphyxia due to poison-induced peripheral respiratory paralysis^9^ ^-^ ^11^.

*In vitro* investigations of venom properties and their effects on the body may aid in understanding this type of progression^12^.
